# Effects of auditory noise intensity and color on the dynamics of upright stance

**DOI:** 10.1038/s41598-024-61186-0

**Published:** 2024-05-08

**Authors:** Sam Carey, Jessica M. Ross, Drew Abney, Ramesh Balasubramaniam

**Affiliations:** 1 Cognitive & Information Sciences, University of California, Merced, 5200 N Lake Road, Merced, CA 95343 USA; 2 Veterans Affairs Palo Alto Healthcare System, Palo Alto, CA USA; 3 Department of Psychiatry and Behavioral Sciences, Stanford University Medical School, Stanford, CA USA; 4 Department of Psychology, University of Georgia, Athens, GA USA

**Keywords:** Postural sway, Auditory feedback, Intensity, White noise, Pink noise, Brown noise, Cognitive neuroscience, Motor control, Sensorimotor processing, Sensory processing

## Abstract

Previous work assessing the effect of additive noise on the postural control system has found a positive effect of additive white noise on postural dynamics. This study covers two separate experiments that were run sequentially to better understand how the structure of the additive noise signal affects postural dynamics, while also furthering our knowledge of how the intensity of auditory stimulation of noise may elicit this phenomenon. Across the two experiments, we introduced three auditory noise stimulations of varying structure (white, pink, and brown noise). Experiment 1 presented the stimuli at 35 dB while Experiment 2 was presented at 75 dB. Our findings demonstrate a decrease in variability of the postural control system regardless of the structure of the noise signal presented, but only for high intensity auditory stimulation.

## Introduction

Postural control has been the subject of scientific interest for many years due to the complexity of the human postural system and its interactions with the environment. Changes in environmental contexts such as: changes in cognitive load^1^, external sensory input^2–6^, or the addition of secondary motor movements^1^ can alter the dynamics of balance. Despite the challenges presented by navigating through an ever-changing environment, the human postural system is capable of adapting to environmental variability quite efficiently. However, the specific processes by which external stimuli are filtered or processed during upright standing are not fully understood.

Postural control is a perceptual motor process that utilizes a continuous stream of sensory input from the auditory, somatosensory, vestibular, and visual systems to maintain a stable body position during standing^7–9^. Human balance relies on this redundancy of sensory input to account for possible changes or perturbations in the expected sensory information from these systems. This process requires the processing of both internal and external sensory information to detect any perturbations that may threaten balance while selecting the necessary motor responses needed to maintain stability^10^. For instance, while the eyes are open, humans rely primarily on visual feedback for balance, but when visual input becomes limited or hindered, we rely more on somatosensory stimulation, such as through a light touch to the finger, to maintain an upright position^11^. Furthermore, studies have demonstrated that additional sensory input through the auditory^7^, somatosensory^5^, vestibular^8^, or visual modalities^9^ can beneficially influence the maintenance of postural stability.

Understanding the sensitivity of the postural system and the ways in which external and internal information influences postural dynamics holds promise for individuals who are at an increased risk of falls. Past work has shown how increases in external sensory information can decrease sway variability and increase stability^2,3,5,7,9,12–14^. However, much remains unknown regarding not only how the presence or absence of new sensory information may influence motor dynamics, but also how the intensity or type of information present may differentially influence these systems. One potential theory to explain why additive sensory information may alter postural dynamics is Stochastic Resonance.

Stochastic Resonance (SR) is a phenomenon observed in nonlinear systems when the addition of noise to a system results in an optimal level of information transfer and an increase in output performance^15,16^. The theory assumes that within a threshold-based system, any underlying information carrying signals within that system can become enhanced through the addition of noise onto the original signal. The addition of noise is assumed to increase the amplitude of the underlying signal, allowing for an increase in the frequency of threshold crossings necessary to send the information the signal is carrying.

SR has been studied in humans in the context of sensory processing^17^, including in auditory^18,19^, visual^20^ and tactile perception^12,21^. In the field of postural control, SR has been used to investigate the impact of noise on sensory information processing and postural stability, with studies showing that the addition of noise can improve postural control in healthy individuals^2,4–6^ and aging populations^3^. Thus, the application of SR to the study of postural control provides a potential avenue for developing interventions to improve balance and reduce the risk of falls in high-risk populations.

One of the major properties of SR is the optimization curve of intensity of the additive noise. An optimal amount of noise results in the maximal enhancement of behavioral performance, whereas further increases in intensity can degrade the output performance of the system of interest^22^. Similarly, too little noise can add no benefit of information transfer^23^. The intensity of noise may play a more critical role than originally thought when the modality of input is considered. Ward and colleagues^24^ distinguished optimal levels of intensity of stimulation during additive noise of the visual, auditory, and tactile modalities. Similarly, during tactile stimulation, Priplata and colleagues^5^ were able to elicit a beneficial response to postural sway with sub-threshold stimulation at the bottom of the foot. Although a major assumption of SR work is that the noise added to the system of interest refers to ‘white noise’, noise can vary in its structure, leading to different ‘forms’ of noise, or more specifically, degrees of noise that are perceivable to the human sensory system. This understanding led us to consider the usefulness of the varying degrees of noise to our sensory system and assess if the structure of the noise signal would affect our motor system differentially.

In signal processing, white noise is a random signal that has equal intensity across all frequencies. Pink noise, on the other hand, has a spectral power density that decreases as frequency increases. As frequency increases, the amplitude of the sound decreases, resulting in a sound that has more bass and less treble. Brownian noise, or brown noise as we will refer to it in this paper, has a type of ‘random walk’ in which the value of the signal at any given point is the sum of the previous value and a normally distributed random value. The potential differences between white, pink, and brown noise may be influential to postural control because they represent distinctive frequencies and distributions of noise signals that may affect the postural control system differently. Understanding the effects of different types of noise on postural control can assist future work to investigate the postural control system’s sensitivity to the structure of sensory inputs and disturbances and how the postural control system adapts to changes in the sensory environment.

In the current study, we examined sway variability during four auditory conditions: silence, white noise, pink noise, and brown noise. All noise conditions were presented with and without visual input. We presented these stimuli across two different experiments, one at a low intensity (35 dB) and one at a high intensity (75 dB). We did this to begin to uncover if the strength and structure of the additive noise are crucial to elicit a reduction in balance variability that has been shown in previous studies^3,4,25^. We hypothesized that different intensities of noise would have the same variability reducing effects on postural sway as seen in past work that used sub-threshold tactile stimulation as the locus of stimulation input^4–6^. Similarly, we expected there to be a reduction in postural sway variability while listening to all three different noise stimuli, when compared to silence. However, we expected the structure of the noise signal to have an impact on the postural dynamics during stimulation. Previous research has shown the influence white noise has on postural sway, but not on how differently structured noise signals may impact sway dynamics. Due to the structure of brown noise having a type of random walk pattern similar to the movement of the Center of Pressure (CoP) during upright standing^26,27^, we expected a reduction in sway variability to be greatest during brown noise stimulation compared to the other noise conditions. Similarly, we expected pink noise to have the same magnitude of effect, or a more beneficial effect compared to white noise due to the lack of structure of white noise and pink noise being closer to brown noise.

Past work by Carey et al.^25^ has shown the ability to induce this effect through the auditory and tactile modalities to similar degrees. By introducing the varying structures of noise, we hoped to be able to understand if frequency matching occurs between the noise signal and our postural control system based on the structure of the signal. If brown noise has the largest effect on balance variability, this suggests that there may be a mechanism at play other than SR. If all three noise signals have the same magnitude of effect on balance, this would support that frequency matching may not have a functional impact on balance variability and that sway reducing effects of noise on balance are better explained with SR or shifting auditory attention.

## Experiment 1

Experiment 1 attempts to expand upon previous work^2,3,25^ on the influence of auditory noise signals on postural sway dynamics by adding noise with varying frequency content compared to previous work that utilized white noise only. This study aimed to replicate the additive noise effect but at a lower intensity than past work (35 dB) and extend to additional types of noise (brown and pink in addition to white). The goal of the study was to establish the effects of differently structured noise signals on postural sway when presented at a low sound intensity.

## Results

### Radial sway

**Figure 1:**
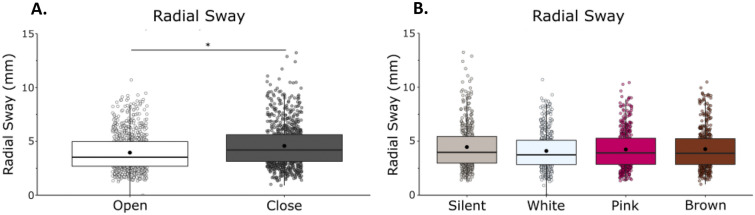
RS was significantly reduced with eyes open, but was unaffected by white noise, pink noise, or brown noise. (**A**) RS in eyes closed/eyes open. (**B**) RS in silent, white, pink, and brown noise conditions. There was no interaction between vision and condition. Box and whiskers plot with the solid black line representing the median, the solid black dot representing the mean, and the extending lines showing the maximum and minimum values. Each individual dot (transparent with a black line around it) represent the Radial Sway value of each of the trial across all the subjects.

Radial sway (RS) was reduced with vision (Fig. 1). We observed a main effect of vision (F(1,21) = 26.36, η = 0.56, *p* = 0.001) with a reduction of RS when eyes were open, and a main effect of condition (F(1,68) = 3.92, η = 0.16, p = 0.0125) on RS (RS; Fig. 1A and 1B) in which there was a reduction in radial sway with the addition of noise. We did not observe any vision by noise condition interactions (F(1,21) = 1.12, η = 0.05, *p* = 0.347).

Bonferroni-corrected post-hoc comparisons were performed to compare the individual noise condition effects on RS when compared to silence and to other noise conditions. Post-hoc comparisons revealed no significant difference between silence (M = 4.46, SD = 2.03) and white noise (M = 4.10, SD = 1.76, *p* = 0.096), pink noise (M = 4.24, SD = 1.78, *p* = 0.109) or brown noise (M = 4.27, SD = 1.83, *p* = 0.568). There was no difference between the stimulation conditions when compared to each other: white and pink (*p* = 0.873), white and brown (*p* = 0.708), pink and brown (*p* = 1.00). The significant main effect of condition is not supported by the post-hoc tests (Supplementary Material: Table 1).

### High-frequency radial sway

**Figure 2:**
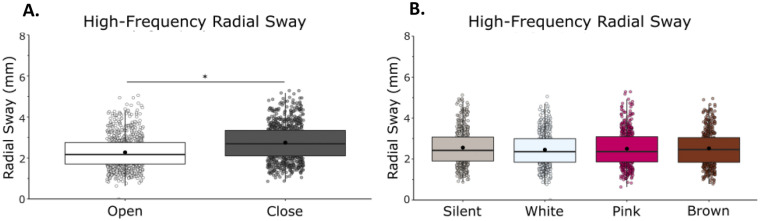
High-frequency (> 0.3 Hz) RS was reduced with eyes open and there were no differences between white noise, pink noise or brown noise. (**A**) High-frequency RS in eyes closed/eyes open. (**B**) High-frequency RS in silent, white, pink, and brown noise conditions. Vision and noise contributed interactively to high-frequency RS. There was also an interaction effect between vision and stimulation. Box and whiskers plot with the solid black line representing the median, the solid black dot representing the mean, and the extending lines showing the maximum and minimum values. Each individual dot (transparent with a black line around it) represent the Radial Sway value of each of the trial across all the subjects.

High-frequency RS was reduced with vision and noise (Fig. 2). We observed a main effect of vision (F(1,21) = 137.76, η = 0.87, *p* = 0.001) with a reduction of RS when eyes were open, and a main effect of condition (F(1,21) = 2.91, η = 0.12, *p* = 0.041) on high-frequency RS, in which there was a reduction in radial sway with the addition of noise (Fig. 2). We observed a vision by noise condition interaction effect, (F(1,21) = 4.38, η = 0.17, *p* = 0.007), which suggests that visual and auditory stimulation contributed interactively to high-frequency sway (Supplementary Material: Table 9).

Bonferroni-corrected post-hoc comparisons were performed to compare the individual noise condition effects on RS when compared to silence and to other noise conditions. When compared to silence (M = 2.56, SD = 0.87), there was no difference in RS when using white noise (M = 2.46, SD = 0.80, *p* = 0.083), pink noise (M = 2.50, SD = 0.85, *p* = 0.618), or brown noise (M = 2.52, SD = 0.79, *p* = 1.00). There was no difference between noise conditions when compared to each other: white and pink (*p* = 1.000), white and brown (*p* = 0.395), pink and brown (*p* = 1.000). The significant main effect of condition is not supported by the post-hoc tests (Supplementary Material: Table 2).

### Low-frequency radial sway

**Figure 3:**
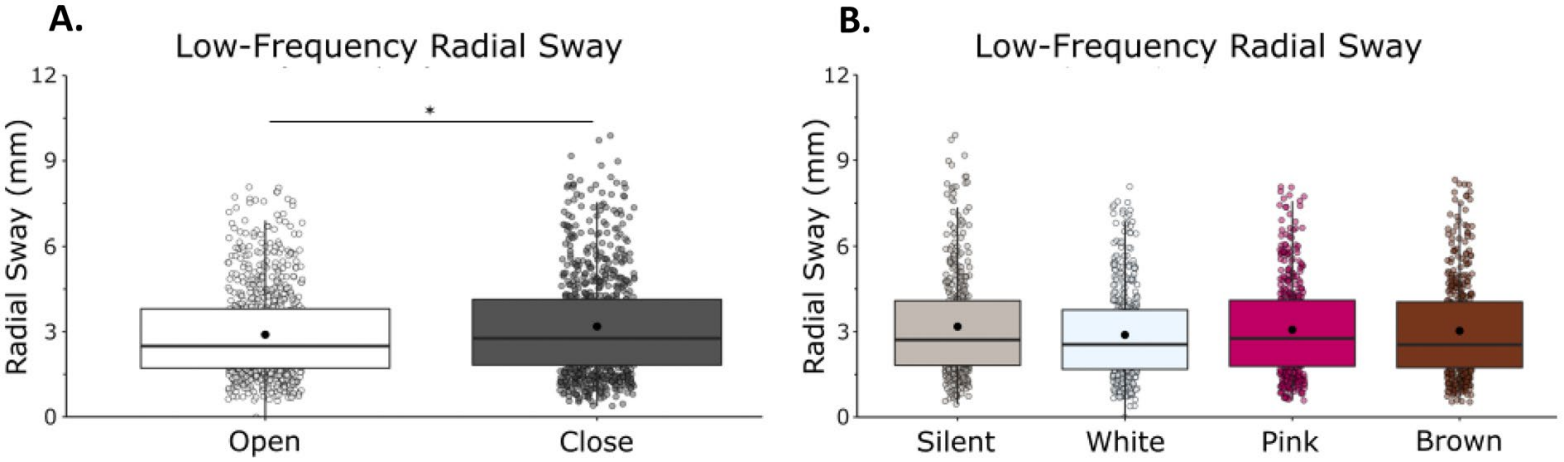
Low-frequency (< 0.3 Hz) RS was reduced with eyes open and there were no differences between white noise, pink noise or brown noise. (**A**) Low-frequency RS in eyes closed/eyes open. (**B**) Low-frequency RS in silent, white, pink, and brown noise conditions. There was no interaction effect between vision and condition. Box and whiskers plot with the solid black line representing the median, the solid black dot representing the mean, and the extending lines showing the maximum and minimum values. Each individual dot (transparent with a black line around it) represent the Radial Sway value of each of the trial across all the subjects.

Low-frequency RS was reduced with vision and with noise (Fig. 3). We observed a main effect of vision (F(1,21) = 6.44, η = 0.23, *p* = 0.019) with a reduction of RS when eyes were open, and a main effect of condition (F(1,21) = 2.79, η = 0.12, *p* = 0.048) on low-frequency RS in which there was a reduction in radial sway with the addition of noise (Fig. 3). We did not observe a vision by noise condition interaction (F(1,21) = 0.6867, η = 0.03, *p* = 0.563).

Bonferroni-corrected post-hoc comparisons were used to compare the individual conditions to each other. There was no difference between silence (M = 3.17, SD = 1.78) and white noise (M = 2.89, SD = 1.58, *p* = 0.208), pink noise (M = 3.07, SD = 1.66, *p* = 1.000) or brown noise (M = 3.03, SD = 1.68, *p* = 1.000). There was no difference between the stimulation conditions when compared to each other: white and pink (*p* = 0.548), white and brown (*p* = 0.993) and pink and brown (*p* = 1.000). The significant main effect of condition is not supported by the post-hoc tests (Supplementary Material: Table 3).

### Detrended fluctuation analysis

**Figure 4:**
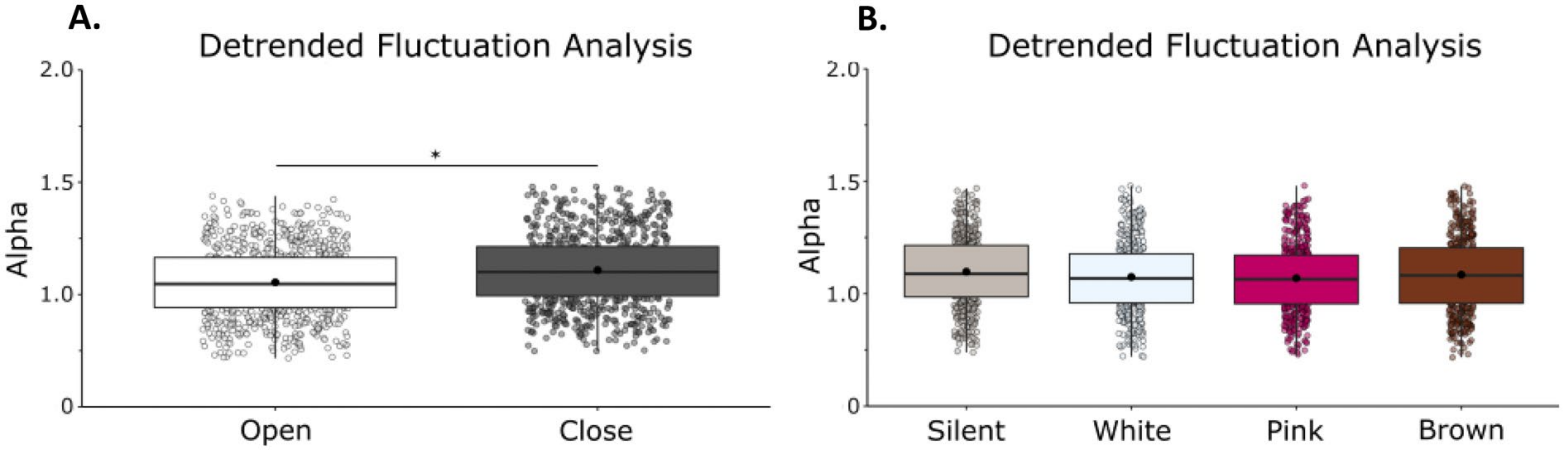
Detrended fluctuation analysis revealed a difference in the random-walk pattern commonly seen in postural sway between eyes open and eyes closed conditions. When eyes were closed there was an increase in alpha within the typical random-walk range. (**A**) Mean α in eyes closed/eyes open (**B**) Mean α in silent, white, pink, and brown noise conditions. Box and whiskers plot with the solid black line representing the median, the solid black dot representing the mean, and the extending lines showing the maximum and minimum values. Each individual dot (transparent with a black line around it) represent the Radial Sway value of each of the trial across all the subjects.

Detrended Fluctuation Analysis showed that RS exhibits anti-persistent fractional Brownian motion (fαm, 1 < α < 1.5). Within this 1–1.5 range, we report differences between conditions in α. We observed a main effect of vision on α (F(1,21) = 39.11, η = 0.65, *p* = 0.001) with a reduction of alpha when eyes were open, (Fig. 4A) but no effect of condition on α (F(1,21) = 2.71, η = 0.11, *p* = 0.052) (Fig. 4B), indicating that sway patterns move in successive steps in random directions (semi-random walk) and tend toward the same direction to a higher degree during eyes open conditions than eyes closed conditions. We did not observe a vision by noise condition interaction (F(1,21) = 0.81, η = 0.04, *p* = 0.492) (Supplementary Material: Table 4).

## Discussion

In Experiment 1, we did not find a convincing effect of the noise stimulation on postural sway variability. All stimuli were presented at 35 dB, which is above the noticeable threshold of the human ear but considered ‘quiet’ when considering that normal conversation happens at around 60–70 dB^28^. We observed significant differences between the visual conditions, with eyes open having a smaller variability of RS than eyes closed, the high- and low-frequency components of RS, and DFA. However, the noise stimulation caused no changes to postural sway dynamics regardless of the noise stimuli structure. Even with the lack of an effect with auditory stimulation, this experiment further validated the importance of visual input on postural sway.

The lack of an effect that the stimulation condition had on postural sway may be explained by the optimization curve of SR. As previously mentioned, an optimal amount of added noise results in the maximal enhancement of behavioral performance, with further increases in intensity degrading the performance of that behavior, and too small of an intensity of noise eliciting no information transfer or changes in behavior^22^. At 35 dB, the stimuli may not be strong enough to elicit any changes and may show that the changes seen in previous work on additive noise^2–6^ may be due to the SR phenomenon and not an attentional focus as commonly posited.

## Experiment 2

In Experiment 1 the intensity of noise stimulation may have been below the effective auditory threshold necessary to elicit the RS reduction reported in previous work with additive auditory noise^2,3,25^. Experiment 2 is a follow up study examining whether there are effects of vision and noise on RS, as in Experiment 1 but at a higher intensity of stimulation (75 dB).

## Results

### Radial sway

**Figure 5:**
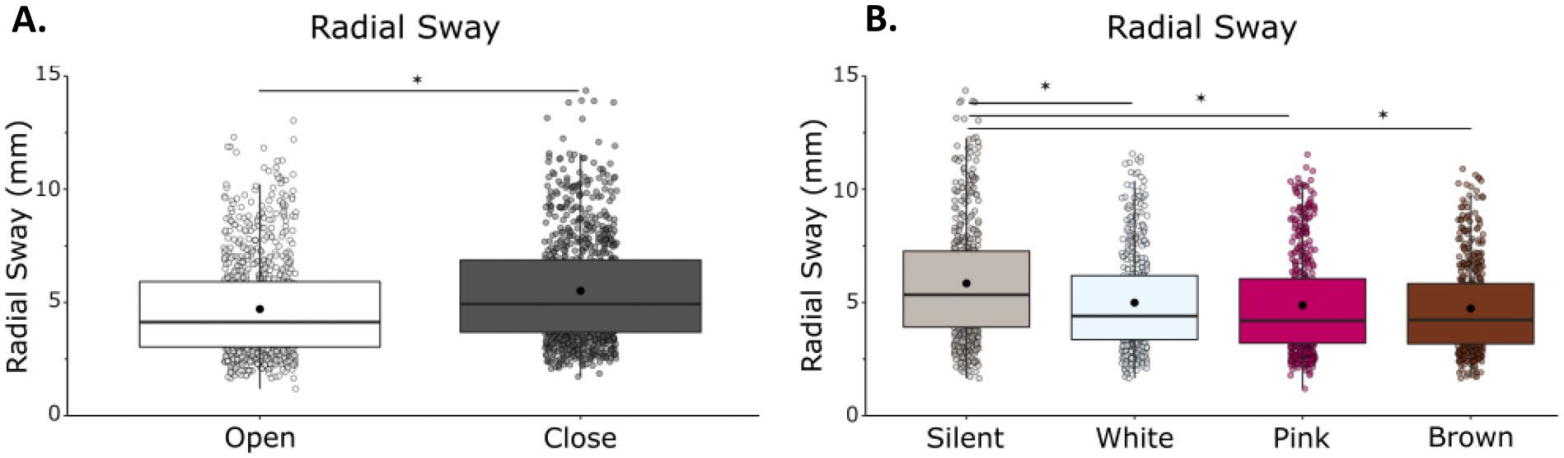
RS is significantly reduced with eyes open, with white noise, pink noise, and brown noise. (**A**) RS in eyes closed/eyes open. (**B**) RS in silent, white, pink, and brown noise conditions. There was no interaction effect between vision and condition. Box and whiskers plot with the solid black line representing the median, the solid black dot representing the mean, and the extending lines showing the maximum and minimum values. Each individual dot (transparent with a black line around it) represent the Radial Sway value of each of the trial across all the subjects.

We observed a main effect of vision (F(1,23) = 39.23, η = 0.63, *p* = 0.001) with a reduction of RS when eyes were open, and a main effect of condition (F(1,23) = 11.94, η = 0.34, *p* = 0.001) on RS, in which there was a reduction in radial sway with the addition of noise (Fig. 5). We did not observe a vision by noise condition interaction (F(1,23) = 1.12, η = 0.05, *p* = 0.346).

Bonferroni-corrected post-hoc comparisons were performed to compare the individual stimulation conditions effects on RS when compared to silence and to other noise conditions. Post-hoc comparisons revealed a significant difference between silence (M = 5.84, SD = 2.58) and white noise (M = 4.98, SD = 2.13, *p* = 0.019), pink noise (M = 4.87, SD = 2.20, *p* = 0.011) and brown noise (M = 4.73, SD = 1.93, *p* = 0.002), confirming the main effect of condition in the ANOVA. We found no difference between stimulation conditions when compared to each other: white and pink (p = 1.00), white and brown (*p* = 0.149), pink and brown (*p* = 0.912) (Supplementary Material: Table 5).

### High-frequency radial sway

**Figure 6:**
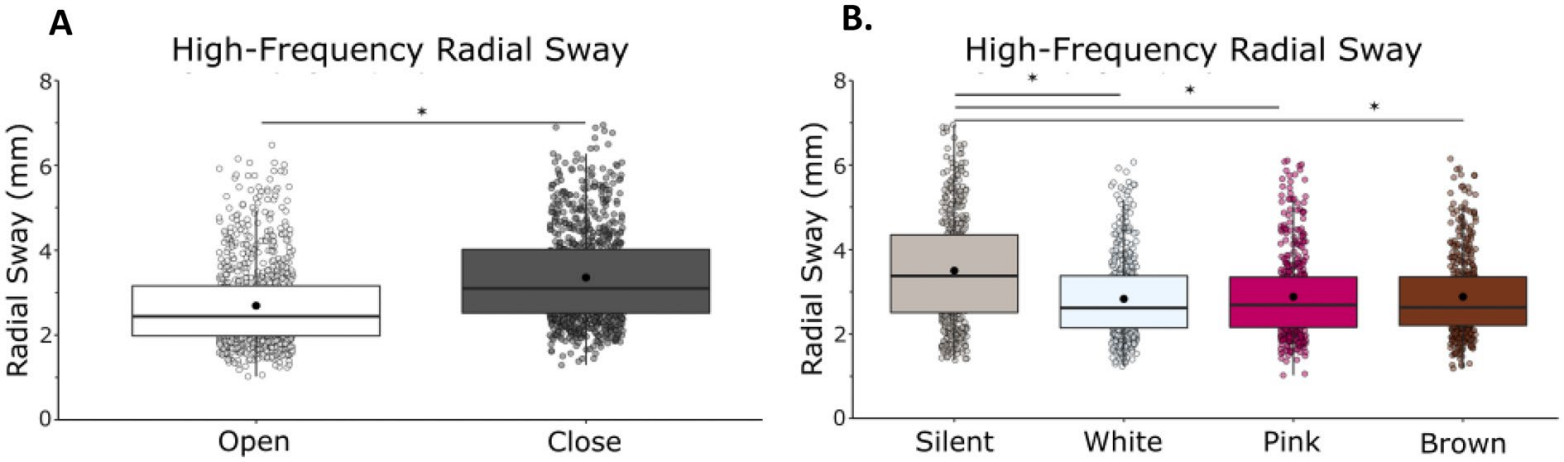
High-frequency (> 0.3 Hz) sway was reduced with eyes open, with white noise, pink noise and brown noise. (**A**) High-frequency RS in eyes closed/eyes open. (**B**) High-frequency RS in silent, white, pink, and brown noise conditions. There was no interaction effect between vision and condition. Box and whiskers plot with the solid black line representing the median, the solid black dot representing the mean, and the extending lines showing the maximum and minimum values. Each individual dot (transparent with a black line around it) represent the Radial Sway value of each of the trial across all the subjects.

We observed a main effect of vision (F(1,23) = 56.98, η = 0.71, *p* = 0.001) with a reduction of RS when eyes were open, and a main effect of condition (F(1,23) = 11.66, η = 0.34, *p* = 0.001) on high-frequency RS, in which there was a reduction in radial sway with the addition of noise (Fig. 6). We did not observe a vision by noise condition interaction (F(1,23) = 2.33, η = 0.09, *p* = 0.082).

Bonferroni-corrected post-hoc comparisons were performed to compare the individual stimulation condition effects on RS when compared to silence and to other noise conditions. Post-hoc comparisons revealed a significant difference between silence (M = 3.50, SD = 1.22) and white noise (M = 2.83, SD = 0.92, *p* = 0.007), pink noise (M = 2.88, SD = 0.95, *p* = 0.018), and brown noise (M = 2.87, SD = 0.88, *p* = 0.009), confirming the main effect of condition in the ANOVA. There was no difference in effect between the noise conditions when compared to each other: white and pink (*p* = 0.568), white and brown (*p* = 1.000), pink and brown (*p* = 1.000) (Supplementary Material: Table 6).

### Low-frequency radial sway

**Figure 7:**
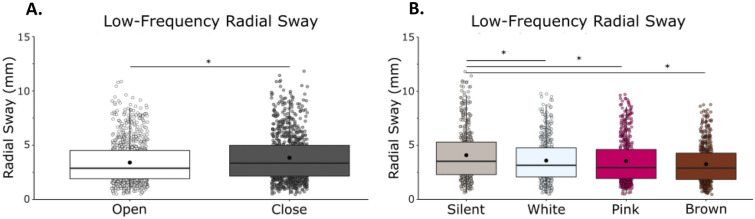
Low-frequency (< 0.3 Hz) sway was reduced with eyes open and with brown noise. (**A**) Low-frequency RS in eyes closed/eyes open. (**B**) Low-frequency RS in silent, white, pink, and brown noise conditions. There was no interaction effect between vision and stimulation. Box and whiskers plot with the solid black line representing the median, the solid black dot representing the mean, and the extending lines showing the maximum and minimum values. Each individual dot (transparent with a black line around it) represent the Radial Sway value of each of the trial across all the subjects.

We observed a main effect of vision (F(1,23) = 17.48, η = 0.43, *p* = 0.001) with a reduction of RS when eyes were open, and a main effect of condition (F(1,23) = 8.86, η = 0.28, *p* = 0.001) on low-frequency RS, in which there was a reduction in radial sway with the addition of noise (Fig. 7A and B). We did not observe a vision and noise condition interaction (F(1,23) = 0.66, η = 0.03, p = 0.576).

Bonferroni-corrected post-hoc comparisons were performed to compare the individual stimulation condition effects on RS when compared to silence and to other noise conditions. Post-hoc comparisons revealed a significant difference between silence (M = 4.08, SD = 2.33) and brown noise (M = 3.26, SD = 1.77, *p* = 0.0016), but no effect of white (M = 3.58, SD = 1.93, *p* = 0.073) or pink noise (M = 3.54, SD = 2.11, *p* = 0.092). There was no difference in effect between the noise conditions when compared to each other: the white and pink (p = 1.000), white and brown (*p* = 0.071) and pink and brown (*p* = 0.124) (Supplementary Material: Table 7).

### Detrended fluctuation analysis

**Figure 8:**
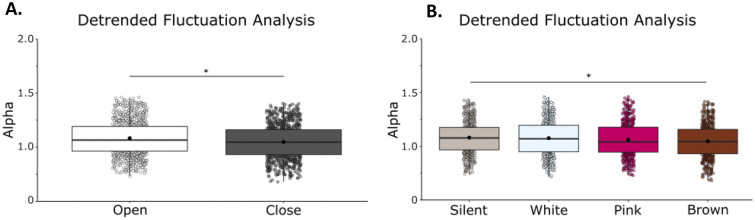
Detrended fluctuation analysis revealed changes with vision and brown noise stimulation in the random-walk pattern of sway. (**A**) Mean α in eyes closed/eyes open (**B**) Mean α in silent, white, pink, and brown noise conditions. Box and whiskers plot with the solid black line representing the median, the solid black dot representing the mean, and the extending lines showing the maximum and minimum values. Each individual dot (transparent with a black line around it) represent the Radial Sway value of each of the trial across all the subjects.

Detrended Fluctuation Analysis showed that our RS data exhibit anti-persistent fractional Brownian motion (fαm, 1 < α < 1.5). We observed a main effect of vision on α (F(1,21) = 13.94, η = 0.38, *p* = 0.001) with a reduction of alpha when eyes were closed, (Fig. 8A) and a main effect of noise condition on α (F(1,21) = 3.97, η = 0.15, *p* = 0.01), in which there was a reduction in radial sway with the addition of noise (Fig. 8B), indicating that with visual input and with noise sway patterns tend toward a more positive correlation than without visual input or noise. We did not observe a vision by noise condition interaction (F(1,21) = 0.16, η = 0.01, *p* = 0.923).

Bonferroni-corrected post-hoc comparisons were performed to compare the individual stimulation condition effects on RS when compared to silence and to other noise conditions. Post-hoc comparisons revealed a significant difference between silence (M = 1.081, SD = 0.149) and brown noise (M = 1.047, SD = 0.158, *p* = 0.027), but not white (M = 1.076, SD = 0.164, p = 1.000) or pink noise (M = 1.061, SD = 0.161, *p* = 0.5644). This finding suggests that there is a shift in the correlational structure of sway specific to brown noise stimulation (Fig. 8B). During brown noise, the scaling exponent becomes closer to α = 1.0 indicating a more negative correlation of the signal, and therefore a more random path of movement. When comparing the stimulation conditions to each other, we found a significant difference between white and brown noise (*p* = 0.027), but no differences between white and pink (*p* = 1.000) or pink and brown (*p* = 1.000) (Supplementary Material: Table 8).

## Discussion

Experiment 2 showed the sway reducing influence that auditory noise stimulation can have on the postural system. Presented at 75 dB, all three noise stimulation conditions caused reductions in the averaged RS and the high- and low-frequency components of RS. The results from Experiment 2 support that postural sway variability is decreased with visual input and with auditory stimulation as similarly found in previous literature^2,3,7,25^.

More interestingly, the DFA scaling exponent was impacted by the structure of the noise presented. Stability can be understood as the co-adjustment of local variability and serial correlational properties^29^. In this study, DFA of sway from participants revealed a lower scaling coefficient (α) when brown noise was introduced and when visual input was available. Higher α indicates more persistence, or more correlation between successive points of sway, and a lower α indicates more anti-persistence in the sway. Anti-persistence can be interpreted as more tightly controlled, or less resistant to changes in CoP displacement direction, which reflects adaptability of the signal to change^30^.

Our results contribute to the knowledge about variability and adaptability by suggesting that the reduction in sway variability with brown noise specifically is accompanied by a potential increase in the adaptability of the postural system. Importantly, however, we emphasize that α was between 1 and 1.5 in all noise stimulation conditions; sway remained anti-persistent and the differences between conditions only appeared between the degree of anti-persistence within this range. Auditory noise stimulation did not interfere with the random walk property of sway, but it may have influenced adaptability as well as variability leading to decreased postural sway.

The reductions seen in RS during auditory noise can be at least partially explained by the SR phenomenon. The theory of SR explains the amplification of information-carrying signals through the addition of noise^16^. This phenomenon relies on a threshold-based system (the postural system), additive noise (auditory stimuli), and an underlying information carrying signal (motor responses of the postural system), all of which were present within this study. However, other possible explanations for the noise effect on postural sway is that there is an increased attentional arousal during stimulation conditions. Our results also suggest that there is frequency matching that could have a functional impact on balance variability and adaptability. More work is needed to determine which mechanisms contribute most to this shift in behavioral output during noise stimulation.

## General discussion

In both experiments, postural variability was significantly reduced with visual input, helping to validate the importance of visual sensory information on the postural control system. The purpose of this paper was to expand upon the literature which shows the beneficial effect auditory noise stimulation can have on postural sway dynamics^2,3,7,25^ by altering the structure of the noise signal and the intensity at which it is presented. Previous work has been able to show how auditory white noise specifically can help increase postural control in younger^2^ and aging populations^3^, as well as the positive effect tactile noise can have on postural control^4–6,25^ but all of these studies have utilized white noise at a single intensity. Within this paper, there was a significant difference in the effect of noise stimulation between the two experiments. During Experiment 1, there was no effect of the noise stimulation on postural sway variability. Although our ANOVAs showed main effects of condition, pairwise comparisons showed no effect between noise and silence. The stimulation was presented at 35 dB, which is above the noticeable threshold of human hearing but is considered ‘quiet’ compared with intensities of around 75 dB, which is slightly higher than human speech^28^. Experiment 2 showed the potential impact that auditory noise can have on our postural sway system. Presented at 75 dB, the noise stimulation caused a reduction in RS when compared to silence, as well as when it was separated into high- and low-frequencies (Table 1 for effect sizes from both experiments). We found no significant differences in RS between the three noise conditions in either experiment. The results from Experiment 2 support that postural sway variability is decreased with visual input and with noise stimulation, regardless of the structure of the noise signal.

**Table 1:** Effect sizes of the comparisons across noise conditions from the ANOVAs of each experiment reported as partial eta squared.

	Radial sway	High-Freq. Sway	Low-Freq. Sway	DFA
Experiment 1 (35 dB)	0.16	0.12	0.12	0.11
Experiment 2 (75 dB)	0.34	0.34	0.28	0.15

An effect size (f) of > 0.4 was considered to reflect a strong effect ^31^.

When observing RS of the high- and low-frequencies of sway, we found that there are equal impacts of the different noise structures on sway. Work by van den Heuvel and colleagues ^32^ and further established by Yeh et al.^33^ showed that sensory feedback can affect these low and high frequency components of sway differentially. The slower timescales of sway, reflecting drift of the inertial mass of the body^34^, are more susceptible to abrupt changes in sensory feedback^32,33,35^. While faster timescales of sway, reflecting small adjustments of the center of mass used to maintain stability, are susceptible to the joint rigidity and muscle activations^36,37^. By separating the components of postural sway into low- and high-frequencies we are able to examine the dynamics of sway more thoroughly to discern if specific dynamics are more heavily influenced by the structure of the noise signals presented. Our results of Experiment 1 support that there was no influence of noise on the separate frequency components of postural sway. However, in Experiment 2 we discovered that additive noise could decrease the RS variability in both the low- and high-frequencies as seen in previous work^2,3,25^. There were no differences between the white, pink, or brown noise in these frequency components, but all three noise signals reduced RS when compared to silence which further supports previous literature showing the beneficial effect of noise on postural sway dynamics^2–6,25^.

The differences in results between Experiment 1 and Experiment 2 were unexpected. However, if we interpret the results while considering the theory of stochastic resonance this effect of intensity is precedented. Past work on SR has shown the influence of the intensity of additive noise for altering the behavioral output of the system under study^22,38^. As previously explained, within the theory of SR added noise helps to enhance the underlying information signal of interest. However, too much added noise to the information signal of interest can cause the signal to become hidden. An optimal amount of added noise results in the maximum enhancement of behavioral output, whereas further increases in the noise intensity only degrade the detectability or information content. Similarly, noise at too low of an intensity may elicit no changes in performance^22^. This relationship between intensity of noise and SR is not linear. As for the interest of this study, Experiment 1 and 2 differed in only one way: the intensity at which the noise stimuli were presented. This difference in noise intensity may explain the differences we see in the results. Too low of a noise intensity may fail to result in alterations in the behavioral output of the system being stimulated. Using a higher intensity elicits the behavioral output expected: a reduction in RS variability and change in the dynamics of postural sway.

Another possible explanation for the noise effect on postural sway is that there is an increased attentional arousal during auditory stimulation, which could lead to a higher level of control in sway. Cluff et al*.*^39^ showed that adding a cognitive task during quiet standing leads to an increase in the automaticity of the postural system and to improvements of stability. However, it has also been shown that passively listening to a single sustained auditory tone does not affect postural sway^14^, so we would not predict that auditory attention in our sustained noise conditions would drive a stabilizing effect in the current experiment. Similarly, if an attentional mechanism was causing this effect, we would expect to see a reduction in RS in both experiments, not just the one of higher intensity. However, it could be the case that lower intensity noise has less attentional demands during the task.

Although the theory of SR at least partially explains our current results, more research is required to determine the specific mechanisms driving this reduction in sway variability. Whether or not these effects are due to SR, attention, or some other mechanism, the findings have profound implications for improving balance in populations at high-risk for falls. One explanation for the minimal effects of structure (white, pink, brown) on the noise effect is that our sensory systems processes and utilize all forms of ‘noise’ in similar manners. Whether it be through the auditory or tactile domain, the resultant behavioral output remains the same regardless of the modality in which the noise is input and processed.

Regarding the arrangement of the SPL meter and headphone during calibration, the SPL was placed at the edge of the headphones compared to the proper methodology of using an artificial ear or dummy head which is common practice within the audiology fields^40^. While we acknowledge the limitations of our headphone calibration procedure, we followed best practices within our resources to ensure the accuracy and reliability of our results given the tools we had available.

## Methods

### Experimental design

The current study included two within-subject experiments that were conducted three months apart. No subject participated in both experiments. The intention was to study how auditory stimulation and the structure of noise signals may influence postural control during standing. First, we tested 3 noise types using a low intensity of 35 dB. We then ran the second experiment at a higher intensity (75 dB) in an attempt to understand whether the intensity of noise amplitude may have influenced the results of Experiment 1. The sound intensities were validated with a Larson Davis cal200 decibel meter. The discomfort of participants for the intensity used was assessed just prior to data collection. To confirm that the intensity was not uncomfortable, we played a sample of noise at the experimental intensity for 30 s and then asked the participant whether they experienced any discomfort. All participants reported no discomfort at the tested intensities. The experiment space where the participants performed the task is a quiet laboratory room 529 cm by 327 cm in size. Within the participant’s visual field, there were 3 motion capture cameras which do not make any noise and a black crosshair on a white wall that the participants were instructed to focus on during standing. There was a negligible amount of machine noise in the room was from the experiment computer, located approximately 8 feet away from the participant.

In each Experiment, the effects of noise during eyes opened and eyes closed on mean RS were modeled across conditions using a two (eyes open vs. eyes closed) × four (silence vs. white vs. pink vs. brown) analysis of variance with repeated measures. A power analysis with a strong effect size (> 0.4) when using a two-way repeated measured ANOVA with two levels (eyes open vs. eyes closed) and four conditions (silence vs. white vs. pink vs. brown) was performed for each study that resulted in an approximate 25 participants needed to observe a significant effect size. Corrections for multiple comparisons were calculated using the Tukey’s method.

### Participants in experiment 1

Twenty-four healthy young adults, 7 male and 17 female, (mean age = 20.61 ± 2.87 years) of varying heights (64.63 ± 5.15 inches) and weights (148.71 ± 25.85 lbs.) were recruited from the University of California, Merced student population. Self-report screeners were used to exclude participants with hearing impairments, arthritis, orthopedic conditions, or neurological disorders^2,3^. No participants reported recent injuries or skeletomuscular disorders, and all could stand unassisted during the experiment. Ethical approval: This study was conducted in accordance with the guidelines set forth by the Institutional Review Board (IRB) at the University of California Merced. The experimental protocol was carried out in accordance with the Declaration of Helsinki and approved by the International Review Board (IRB) of the University of California (IRB approval code: UCM14-0008), ensuring compliance with ethical standards and participant welfare throughout the study. Informed consent was obtained from all participants prior to testing.

Participants were instructed to stand on a force platform in a relaxed, comfortable standing position with their arms at their sides while wearing headphones (Sennheiser HD 280 pro) through which the auditory stimuli were presented. Participants were periodically reminded of the instructions between trials and during breaks throughout the duration of the study. The headphones intensity was set on the desktop and were calibrated with a sound level meter. The headphones were closed but not noise cancelling. There was 42.5 dB of ambient noise within the lab during collection, however the headphones used have an ambient noise attenuation of ≤ 32 dB and participants wore the headphones for the entire duration of the study, including during silent conditions. Participants were instructed to keep their eyes fixated on a black crosshair stimulus posted on the wall 229 cm in front of them at approximately eye level for the eyes-open trials and to keep their head facing forward and eyes closed during eyes closed trials^2,3^. The average session was 45 min. Participants received a short break every 10 trials (~ 7 min) to step off the force plate and stretch and a longer break halfway through the experiment to sit down and rest for 5 min. Similarly, to test for any possible adaptation effects during the trials, the first and last ten seconds of the trials were compared. No changes in postural variability were found between the beginning and end of the trials, which confirms that there were no adaptation related postural changes during the period of auditory stimulation (as shown in supplementary table 10).

The noise (silence, white, pink, brown) and visual input (eyes open, eyes closed) conditions were presented in a randomized order. There was a total of 80 trials, 20 trials for each noise condition, 10 with eyes opened and 10 with eyes closed. The trials lasted 20 s and were accompanied by silence, auditory white noise, auditory pink noise, or auditory brown noise. The noise was presented at an intensity of 35 dB SPL in Experiment 1 and 75 dB SPL in Experiment 2. CoP was sampled at 200 Hz with an AMTI Force and Motion platform (Optima BP400600-2000 GEN 5; AMTI Force and Motion, Watertown, MA, USA). All data for each subject were collected in a single session. The auditory noise stimuli were algorithmically generated using MATLAB, with the white noise composed of random signals with a constant spectral density. Similarly, pink noise was generated such that the power spectral density is inversely proportional to the frequency of the signal (frequency density proportional to 1/*f*) and Brown noise with a spectral density that is inversely proportional to *f*^*2*^*,* meaning it has higher intensities at lower frequencies (frequency density proportional to 1/* f*^*2*^)^41^. Participants were exposed to the noise stimuli prior to the experiment to verify that the intensity was not uncomfortable for them. No participants reported discomfort at these intensities.

### Participants in experiment 2

Twenty-two healthy young adults, 9 male and 13 female, (mean age = 21.96 ± 3.42 years) of varying heights (65.56 ± 3.48 inches) and weights (141.76 ± 27.28 lbs.) were recruited from the University of California, Merced student population. Self-report screenings were used to exclude participants with hearing impairments, arthritis, orthopedic conditions, or neurological disorders^2,3^. No participants reported recent injuries or skeletomuscular disorders, and all could stand unassisted during the experiment. Ethical approval: This study was conducted in accordance with the guidelines set forth by the Institutional Review Board (IRB) at the University of California Merced. The experimental protocol was reviewed and approved by the IRB (IRB approval code: UCM14-0008), ensuring compliance with ethical standards and participant welfare throughout the study. Informed consent was obtained from all participants prior to testing.

### Analysis

The CoP of each condition was analyzed using custom scripts in MATLAB (MathWorks, Natick, MA, USA). The first 4 s of each trial were removed to eliminate any potential startle response that participants might have had to stimulus onset. Radial sway (RS) of the CoP was calculated for each sample (i) using the anterior–posterior (A-P; x) and medial–lateral (M-L; y) components of sway (Eq. 1) following^42^:

1 $${RS}_{i}= \sqrt{{x}_{i}^{2}+ {y}_{i}^{2}}$$

Average RS was calculated for each trial and was used to assess bidirectional variability in CoP during the trials^42^. Although RS is not a direct metric of stability, it utilizes the multidirectional variability of sway to offer a more robust understanding of the sway dynamics which may lead to stability^42^. Trial outliers were determined as trials with averages of $\pm 2$ standard deviations from that subject’s mean within condition and were removed. In Experiment 1, 203 of the total 5280 trials were removed, resulting in a removal rate of 4%. In Experiment 2, 295 of the total 5,760 trials were removed resulting in a 5% removal rate.

In each Experiment , the effects of noise during eyes opened and eyes closed on mean RS were modeled across conditions using a two (eyes open vs. eyes closed) × four (silence vs. white vs. pink vs. brown) analysis of variance with repeated measures.

The analysis was then repeated using the filtered high and low frequency RS separately to assess changes in slower and faster timescales of postural control (following the methods of 33 and 35). Postural sway is naturally oscillatory and is composed of two primary timescales of oscillation^33^. Low-frequency oscillations are typically considered to reflect feedback-based corrective responses, where high-frequency oscillations are considered open-loop exploratory processes^35^. We used low- and high-pass Butterworth filtering routines, as in Yeh et al.^35^, to decompose sway into low (< 0.3 Hz) and high (> 0.3 Hz) frequency sway. The filter cutoff was chosen based on van den Heuvel et al.^32^ and Jeka et al.^13^ to separate into sensory feedback-related sway and spontaneous/exploratory sway.

Detrended fluctuation analysis (DFA) was used to assess the sway dynamics over time while under different stimulation conditions ^27,43^. DFA is used to study the behavior of the timeseries of CoP. This analysis, first introduced by Peng et al.^44^, is a scaling analysis method that provides a scaling exponent $\alpha$, which offers information about the correlational properties of the CoP signal. When the DFA value exists between 1 < $\alpha$  < 1.5, the postural sway is considered antipersistent. This means that the sway moves in successive steps in random directions (a semi-random walk) and does not trend toward the same direction. Anti-persistence can be interpreted as more tightly controlled, or less resistance to changes in CoP displacement direction, which reflects adaptability to of the signal to change. The scaling exponent $\alpha$ includes the information concerning the correlation properties of the signal: $\alpha =1.5$ is characteristic of an uncorrelated random series (white noise), while the signal presents positive correlations if $\alpha >1.5$ and negative correlations if $\alpha <1.5$. Antipersistent RS dynamics are commonly described in healthy postural sway.

To calculate DFA, the series is first integrated and then detrended within each considered interval. Finally, the standard deviation of the integrated and detrended series is computed. The scaling exponent is then estimated as the slope of the double-logarithmic plot of standard deviation, as a function of interval length^43^. This analysis was completed as in (Blázquez et al.^29^) using the same parameters. See Blázquez et al.^29^ and Delignières et al.^43^ for more details on the DFA method. Our data was passed through a DFA algorithm^45^ to calculate the $\alpha$ of each trial within each condition and then averaged across trials within the same conditions. The calculation is as follows ^46^:

Step 1: Briefly, the CoP series (of total length N) is integrated as follows:

$$y\left(k\right)={\sum }_{t=1}^{k}[{\text{X}}\left(t\right)-\overline{{\text{x}} }]$$

Step 2: The integrated time series y(*k)* is divided into sub-sequences of equal length n, a least-squared line is fit to the data (representing the trend in that box).

Step 3: We detrend the integrated time series y*(k)* by subtracting the local trend y_n_(*k*) in each box. The root-mean squared fluctuation of this integrated and detrended time series is calculated as follows:

$$F(n)=\sqrt{\frac{1}{N}+{\sum }_{k=1}^{N}{[{\text{y}}\left({\text{k}}\right)-{y}_{n}\left(k\right)]}^{2}}$$

The computation is repeated over all time scales (box sizes) to provide a relationship between F(n), the average fluctuation as a function of box size, and box size n (such as the number of positions in a box that is the window of observation). Typically, F(n) will increase with box size n.

We assessed the normality of the data distribution by examining a Quantile–Quantile (Q-Q) plot. The Q-Q plot compared the observed quantiles of the data against the quantiles expected under a normal distribution. The plot supported that our data have a normal distribution. This graphical method provides a robust visualization of the data distribution and allows for a qualitative assessment of normality. Similarly, we calculated skewness for the radial sway data in which all other variables were calculated from. Our assessment led to a skewness value of 0.5815931 for high intensity Radial Sway and 0.702614 for low intensity Radial sway.

### Supplementary Information

Supplementary Tables.

## Data Availability

The datasets generated during and/or analyzed during the current study are available from the corresponding author on reasonable request.
