# Supplementary material for: Effects of auditory noise intensity and color on the dynamics of upright stance
Source: Sci Rep. 2024 May 8;14:10518. doi: 10.1038/s41598-024-61186-0 (PMC11076473; doi:10.1038/s41598-024-61186-0)
Supplement: Supplementary file 1 — Supplementary Tables. [file 41598_2024_61186_MOESM1_ESM.docx]

**Supplementary Information**

**Effects of auditory noise intensity and color on the dynamics of upright stance**

*Sam Carey^1^, Jessica M. Ross^2,3^, Drew Abney^4^, Ramesh Balasubramaniam^1^

1 Cognitive & Information Sciences, University of California, Merced, CA, United States

2 Veterans Affairs Palo Alto Healthcare System and the Sierra Pacific Mental Illness, Research, Education, and Clinical Center, Palo Alto, CA, United States

3 Department of Psychiatry and Behavioral Sciences, Stanford University Medical Center, Stanford, CA, United States

4 Department of Psychology, University of Georgia, Athens, GA, United States

Supplemental Table 1.

| **Low-Intensity Radial Sway** | | | | | |
| --- | --- | --- | --- | --- | --- |
| **Contrast** | **Estimate** | **SE** | **DF** | ***t* ratio** | ***p* value** |
| Silent-White | 0.3959 | 0.1511 | 21 | 2.621 | 0.0958 |
| Silent-Pink | 0.2276 | 0.0889 | 21 | 2.561 | 0.1093 |
| Silent-Brown | 0.2055 | 0.1174 | 21 | 1.750 | 0.5680 |
| White-Pink | -0.1683 | 0.1113 | 21 | -1.512 | 0.8732 |
| White-Brown | -0.1904 | 0.1168 | 21 | -1.630 | 0.7084 |
| Pink-Brown | -0.0221 | 0.1006 | 21 | -0.220 | 1.0000 |

Supplemental Table 2.

| **Low-Intensity High-Frequency Radial Sway** | | | | | |
| --- | --- | --- | --- | --- | --- |
| **Contrast** | **Estimate** | **SE** | **DF** | ***t* ratio** | ***p* value** |
| Silent-White | 0.1091 | 0.0406 | 21 | 2.689 | 0.0825 |
| Silent-Pink | 0.0688 | 0.0404 | 21 | 1.705 | 0.6181 |
| Silent-Brown | 0.0463 | 0.0417 | 21 | 1.108 | 1.0000 |
| White-Pink | -0.0404 | 0.0349 | 21 | -1.155 | 1.0000 |
| White-Brown | -0.0629 | 0.0324 | 21 | -1.941 | 0.3951 |
| Pink-Brown | -0.0225 | 0.0354 | 21 | -0.636 | 1.0000 |

Supplemental Table 3.

| **Low-Intensity Low-Frequency Radial Sway** | | | | | |
| --- | --- | --- | --- | --- | --- |
| **Contrast** | **Estimate** | **SE** | **DF** | ***t* ratio** | ***p* value** |
| Silent-White | 0.3286 | 0.1456 | 21 | 2.257 | 0.2088 |
| Silent-Pink | 0.1174 | 0.0837 | 21 | 1.403 | 1.0000 |
| Silent-Brown | 0.1602 | 0.1136 | 21 | 1.411 | 1.0000 |
| White-Pink | -0.2111 | 0.1194 | 21 | -1.769 | 0.5488 |
| White-Brown | -0.1683 | 0.1172 | 21 | -1.437 | 0.9931 |
| Pink-Brown | 0.0428 | 0.1023 | 21 | 0.418 | 1.0000 |

Supplemental Table 4.

| **Low-Intensity Detrended Fluctuation Analysis** | | | | | |
| --- | --- | --- | --- | --- | --- |
| **Contrast** | **Estimate** | **SE** | **DF** | ***t* ratio** | ***p* value** |
| Silent-White | 0.02390 | 0.01078 | 21 | 2.216 | 0.2272 |
| Silent-Pink | 0.02916 | 0.01005 | 21 | 2.902 | 0.0512 |
| Silent-Brown | 0.01730 | 0.01063 | 21 | 1.628 | 0.7109 |
| White-Pink | 0.00526 | 0.01068 | 21 | 0.492 | 1.0000 |
| White-Brown | -0.00660 | 0.01293 | 21 | -0.510 | 0.0313 |
| Pink-Brown | -0.01186 | 0.00996 | 21 | -1.191 | 1.0000 |

Supplemental Table 5.

| **High-Intensity Radial Sway** | | | | | |
| --- | --- | --- | --- | --- | --- |
| **Contrast** | **Estimate** | **SE** | **DF** | ***t* ratio** | ***p* value** |
| Silent-White | 0.8567 | 0.2612 | 23 | 3.280 | 0.0197 |
| Silent-Pink | 0.9320 | 0.2641 | 23 | 3.530 | 0.0108 |
| Silent-Brown | 1.0949 | 0.2603 | 23 | 4.206 | 0.0020 |
| White-Pink | 0.0753 | 0.1216 | 23 | 0.619 | 1.0000 |
| White-Brown | 0.2382 | 0.0993 | 23 | 2.399 | 0.1496 |
| Pink-Brown | 0.1630 | 0.1100 | 23 | 1.482 | 0.9122 |

Supplemental Table 6.

| **High-Intensity High-Frequency Radial Sway** | | | | | |
| --- | --- | --- | --- | --- | --- |
| **Contrast** | **Estimate** | **SE** | **DF** | ***t* ratio** | ***p* value** |
| Silent-White | 0.6448 | 0.1751 | 23 | 3.682 | 0.0074 |
| Silent-Pink | 0.5624 | 0.1700 | 23 | 3.307 | 0.0184 |
| Silent-Brown | 0.5976 | 0.1671 | 23 | 3.576 | 0.0096 |
| White-Pink | -0.0823 | 0.0472 | 23 | -1.743 | 0.5685 |
| White-Brown | -0.0472 | 0.0376 | 23 | -1.255 | 1.0000 |
| Pink-Brown | 0.0351 | 0.0561 | 23 | 0.626 | 1.0000 |

Supplemental Table 7.

| **High-Intensity Low-Frequency Radial Sway** | | | | | |
| --- | --- | --- | --- | --- | --- |
| **Contrast** | **Estimate** | **SE** | **DF** | ***t* ratio** | ***p* value** |
| Silent-White | 0.4990 | 0.184 | 23 | 2.717 | 0.0738 |
| Silent-Pink | 0.5103 | 0.195 | 23 | 2.615 | 0.0928 |
| Silent-Brown | 0.7976 | 0.186 | 23 | 4.294 | 0.0016 |
| White-Pink | 0.0113 | 0.131 | 23 | 0.086 | 1.0000 |
| White-Brown | 0.2986 | 0.109 | 23 | 2.736 | 0.0706 |
| Pink-Brown | 0.2873 | 0.116 | 23 | 2.482 | 0.1247 |

Supplemental Table 8.

| **High-Intensity Detrended Fluctuation Analysis** | | | | | |
| --- | --- | --- | --- | --- | --- |
| **Contrast** | **Estimate** | **SE** | **DF** | ***t* ratio** | ***p* value** |
| Silent-White | 0.00597 | 0.01135 | 23 | 0.527 | 1.0000 |
| Silent-Pink | 0.01934 | 0.01108 | 23 | 1.747 | 0.5644 |
| Silent-Brown | 0.03364 | 0.01068 | 23 | 3.150 | 0.0269 |
| White-Pink | 0.01337 | 0.01140 | 23 | 1.173 | 1.0000 |
| White-Brown | 0.02766 | 0.00896 | 23 | 3.087 | 0.0313 |
| Pink-Brown | 0.01430 | 0.01011 | 23 | 1.415 | 1.0000 |

Supplemental Table 9.

| **Low-Intensity High-Frequency Vision/Condition Interaction** | | | | | |
| --- | --- | --- | --- | --- | --- |
| **Contrast** | **Estimate** | **SE** | **DF** | ***t* ratio** | ***p* value** |
| Silent Open -White Open | 0.1601 | 0.0580 | 21 | 2.763 | 0.3267 |
| Silent Open - Pink Open | 0.1140 | 0.0639 | 21 | 1.785 | 1.0000 |
| Silent Open - Brown Open | 0.1775 | 0.0647 | 21 | 2.742 | 0.3418 |
| Silent Open - Silent Closed | -0.3664 | 0.0624 | 21 | -5.871 | 0.0002 |
| Silent Open - White Closed | -0.3082 | 0.0525 | 21 | -5.866 | 0.0002 |
| Silent Open - Pink Closed | -0.3428 | 0.0663 | 21 | -5.167 | 0.0011 |
| Silent Open - Brown Closed | -0.4513 | 0.0696 | 21 | -6.481 | 0.0001 |
| White Open - Pink Open | -0.0461 | 0.0392 | 21 | -1.176 | 1.0000 |
| White Open - Brown Open | 0.0174 | 0.0472 | 21 | 0.369 | 1.0000 |
| White Open - Silent Closed | -0.5265 | 0.0639 | 21 | -8.243 | <.0001 |
| White Open - White Closed | -0.4683 | 0.0487 | 21 | -9.620 | <.0001 |
| White Open - Pink Closed | -0.5029 | 0.0647 | 21 | -7.775 | <.0001 |
| White Open - Brown Closed | -0.6114 | 0.0672 | 21 | -9.093 | <.0001 |
| Pink Open - Brown Open | 0.0635 | 0.0494 | 21 | 1.284 | 1.0000 |
| Pink Open - Silent Closed | -0.4804 | 0.0493 | 21 | -9.748 | <.0001 |
| Pink Open - White Closed | -0.4222 | 0.0461 | 21 | -9.162 | <.0001 |
| Pink Open - Pink Closed | -0.4569 | 0.0575 | 21 | -7.946 | <.0001 |
| Pink Open - Brown Closed | -0.5654 | 0.0550 | 21 | -10.282 | <.0001 |
| Brown Open - Silent Closed | -0.5439 | 0.0685 | 21 | -7.935 | <.0001 |
| Brown Open - White Closed | -0.4857 | 0.0517 | 21 | -9.386 | <.0001 |
| Brown Open - Pink Closed | -0.5203 | 0.0747 | 21 | -6.966 | <.0001 |
| Brown Open - Brown Closed | -0.6288 | 0.0727 | 21 | -8.649 | <.0001 |
| Silent Closed - White Closed | 0.0582 | 0.0515 | 21 | 1.129 | 1.0000 |
| Silent Closed - Pink Closed | 0.0235 | 0.0529 | 21 | 0.445 | 1.0000 |
| Silent Closed - Brown Closed | -0.0850 | 0.0491 | 21 | -1.729 | 1.0000 |
| White Closed - Pink Closed | -0.0346 | 0.0521 | 21 | -0.665 | 1.0000 |
| White Closed - Brown Closed | -0.1432 | 0.0493 | 21 | -2.903 | 0.2382 |
| Pink Closed - Brown Closed | -0.1085 | 0.0506 | 21 | -2.144 | 1.0000 |

Supplemental Table 10.

| **3-way ANOVA of Radial Sway** | | | | | |
| --- | --- | --- | --- | --- | --- |
|  | **DF** | **Sum Sq** | **Mean Square** | ***F* Value** | ***p* value** |
| Intensity | 1 | 625 | 624.7 | 147.069 | <2e-16*** |
| Eyes | 1 | 450 | 449.7 | 105.869 | <2e-16*** |
| Condition | 3 | 263 | 87.6 | 20.630 | 3.02e-13*** |
| Intensity:Eyes | 1 | 7 | 7.4 | 1.754 | 0.186 |
| Intensity:Condition | 3 | 106 | 35.4 | 8.335 | 1.60e-05*** |
| Eyes:Condition | 3 | 17 | 5.7 | 1.337 | 0.260 |
| Intensity:Eyes:Condition | 3 | 6 | 2.0 | 0.475 | 0.699 |
| Residuals | 3500 | 14866 | 4.2 |  |  |

Supplemental Table 11.

| **Comparison of First 10 and Last 10 seconds of each trial** | | | | | |
| --- | --- | --- | --- | --- | --- |
| **Contrast** | **Estimate** | **SE** | **DF** | ***t* ratio** | ***p* value** |
| First 10 – Last 10 | 1.88e-14 | 0.0603 | 2870 | 0.000 | 1.000 |

**Degrees-of-freedom method: kenward-roger*
